# Application of fungal copper carbonate nanoparticles as environmental catalysts: organic dye degradation and chromate removal

**DOI:** 10.1099/mic.0.001116

**Published:** 2021-12-09

**Authors:** Feixue Liu, Dinesh Singh Shah, Laszlo Csetenyi, Geoffrey Michael Gadd

**Affiliations:** ^1^​ Geomicrobiology Group, School of Life Sciences, University of Dundee, Dundee, UK; ^2^​ Division of Cell Signalling and Immunology, School of Life Sciences, University of Dundee, Dundee, UK; ^3^​ Concrete Technology Group, Department of Civil Engineering, University of Dundee, Dundee, UK; ^4^​ State Key Laboratory of Heavy Oil Processing, Beijing Key Laboratory of Oil and Gas Pollution Control, College of Chemical Engineering and Environment, China University of Petroleum, Beijing, PR China

**Keywords:** biosynthesis, bioremediation, copper nanoparticles, fungi, *Neurospora crassa*

## Abstract

Biomineralization is a ubiquitous process in organisms to produce biominerals, and a wide range of metallic nanoscale minerals can be produced as a consequence of the interactions of micro-organisms with metals and minerals. Copper-bearing nanoparticles produced by biomineralization mechanisms have a variety of applications due to their remarkable catalytic efficiency, antibacterial properties and low production cost. In this study, we demonstrate the biotechnological potential of copper carbonate nanoparticles (CuNPs) synthesized using a carbonate-enriched biomass-free ureolytic fungal spent culture supernatant. The efficiency of the CuNPs in pollutant remediation was investigated using a dye (methyl red) and a toxic metal oxyanion, chromate Cr(VI). The biogenic CuNPs exhibited excellent catalytic properties in a Fenton-like reaction to degrade methyl red, and efficiently removed Cr(VI) from solution due to both adsorption and reduction of Cr(VI). X-ray photoelectron spectroscopy (XPS) identified the oxidation of reducing Cu species of the CuNPs during the reaction with Cr(VI). This work shows that urease-positive fungi can play an important role not only in the biorecovery of metals through the production of insoluble nanoscale carbonates, but also provides novel and simple strategies for the preparation of sustainable nanomineral products with catalytic properties applicable to the bioremediation of organic and metallic pollutants, solely and in mixtures.

## Introduction

Nanoscience is a rapidly developing field that covers a wide-range of applications in different areas of science and technology, and research on the synthesis of biogenic nanoparticles is receiving increasing attention. Compared with bulk materials, the special properties and characteristics of nanomaterials make them relevant for a variety of applications, e.g. electronics, photonics, catalysis, photography, material coatings, biotechnology, medicine, pharmacology and textile manufacture [1–6]. In order to find methods of synthesizing nanoparticles at a lower energetic and environmental cost, a variety of organisms (e.g. bacteria, filamentous fungi, yeasts and plants) have been harnessed to produce nanoparticles [7–10]. For the synthesis of nanoparticles using fungi, both living and dead mycelium [11] and biomass-free spent fungal growth medium [12] have been used to react with metal salt solutions to successfully produce nanoparticles. In some cases, biogenic nanomaterials can exhibit unique properties and morphologies that are unobtainable by conventional chemical synthesis. The synthesizing process is also low-cost and environmentally benign, with straightforward downstream biomass waste processing. Since proteins and other biomolecules involved in their synthesis can act as capping agents and improve stability, this avoids the use of hazardous capping ligands and residual toxins produced during chemical nanoparticle synthesis [13].

Biogenic approaches have made a significant contribution to the production of metallic, metallic oxide and bimetallic nanoparticles [14, 15]. There has been a surge of interest in the synthesis of copper-based nanostructures using biological systems due to their remarkable catalytic efficiency and antibacterial properties [16]. Copper is also cheaper than some other elements that have been extensively studied as nanoparticles, e.g. silver and gold, suggesting the production of Cu nanoparticles could be cost-effective [17]. *Neurospora crassa* is a well-studied fungal species, which can be harnessed to synthesize a wide range of catalytically active metal-containing nanoparticles, and the production of extracellular metal nanoparticles, including Cd, Cu and Fe, by *N. crassa* has been reported [18–21]. In a urea-containing medium, carbonate ions are released from urea hydrolysis by the enzyme urea amidohydrolase (urease) of *N. crassa*, and various metal carbonate minerals can be precipitated via the reaction of metal ions and carbonate ions under alkaline conditions [18]. This can be a straightforward and simple process for producing Cu-bearing nanoparticles (CuNPs) although potential applications, such as the use of fungal-synthesized CuNPs as environmental catalysts for pollutant remediation, remain to be tested.

Some applications of Cu-based nanoparticles in environment chemistry have been reported previously [22, 23]. They can offer the potential to remediate a variety of pollutants, including various toxic metals and organic contaminants. Copper can also be sourced from waste processes and may, therefore, provide suitable raw materials for the synthesis of biogenic CuNPs and/or co-remediation of more than one contaminant. In this research, the use of CuNPs to remediate two model pollutants, an organic pollutant, methyl red, and a redox-active toxic metal, chromium (VI), is demonstrated. Methyl red is classed among the azo dyes that are an important class of synthetic organic compounds bearing the functional group (−*N*=N−). Azo dyes are widely used in textile and printing industries [24], and the large-scale production and application of dyes, including azo dyes, can cause considerable environmental problems. The dyes released in the environment in industrial effluents can disturb the natural growth activity of aquatic organisms [25] by affecting photosynthesis and decreasing the amount of dissolved oxygen [26, 27]. Chromium contamination of natural sediments and waters is linked to industrial processes, mainly metal plating, alloy production, dye and pigment production, and leather and wood preservation (EPA, 2010). Chromium occurs in the Cr(III) and Cr(VI) oxidation states, and Cr(VI) is recognized as highly toxic and carcinogenic, while Cr(III) has a relatively lower toxicity. The oxidation states of Cr(VI), as chromate (CrO_4_^2-^) and dichromate (Cr_2_O_7_^2-^), show very high mobility in natural environments, which increases potential health risks to living organisms, including humans. Compared with Cr(VI), the Cr(III) oxidation state forms insoluble oxyhydroxides, that have a strong affinity for adsorption onto the surfaces of soil minerals. Therefore, the reduction of highly mobile Cr(VI) to immobile Cr(III) has become a major focus for controlling chromium contamination [28].

Various physico-chemical and biological approaches have been developed to remove pollutants from aqueous solution, such as adsorption, electrochemical or chemical oxidation, and biological aerobic/anaerobic decomposition [25, 29]. In this study, the effective degradation of methyl red (MR) under neutral conditions by bio-synthesized CuNPs with promising reusability is reported. In addition, the removal of Cr(VI) by CuNPs via adsorption and reduction is also demonstrated. In view of the considerable concerns about the biological effects of nanoparticles that have arisen with the development of nanoscience and nanotechnology, the cytotoxicity of CuNPs was also studied. Our work provides further understanding of relevance to potential applications of metal nanoparticles that are produced from green and economic biosynthesis, and highlights their relevance for design of co-remediation techniques for mixed metallic and organic pollutants.

## Methods

### Biomineralization of CuNPs

The experimental fungus used in this study was *N. crassa* [FGSC: 2489, Fungal Genetics Stock Centre (FGSC), Manhattan, KS, USA]. After 3 days of growth on malt extract agar (MEA, Lab M, UK) in 90 mm diameter Petri dishes at 25 °C in the dark, *N. crassa* was inoculated into a urea-modified AP1 liquid medium (pH=5.5) with 10×5 mm diameter discs cut from the agar plates using a sterile cork borer (5 mm diameter). The AP1 liquid medium consists of 2% (w/v) ᴅ-glucose (Merck, USA), 40 mM urea (Sigma-Aldrich, USA), 4 mM K_2_HPO_4_∙3H_2_O (Sigma-Aldrich, USA), 0.8 mM MgSO_4_∙7H_2_O (Sigma-Aldrich, USA), 0.2 mM CaCl_2_∙6H_2_O (Sigma-Aldrich, USA), 1.7 mM NaCl (Sigma-Aldrich, USA), 9×10^−3^ mM FeCl_3_∙6H_2_O (Sigma-Aldrich, USA) and trace metals 0.014 mM ZnSO_4_∙7H_2_O (VWR, USA), 0.018 mM MnSO_4_∙4H_2_O (Sigma-Aldrich, USA) and 1.6×10^−3^ mM CuSO_4_∙5H_2_O (VWR, USA) [30]. After 3 days of growth in complete AP1 medium, fungal biomass was collected and washed twice in sterile Milli-Q water by centrifugation (×4000 ***g***, 30 min), and then incubated in sterile phosphate-free AP1 medium for 12 days at 125 r.p.m., 25 °C in the dark. CuNPs were produced following previous protocols [31, 32]. Briefly, biomass-free *N. crassa* spent culture media was collected by centrifugation (×4000 ***g***, 30 min) after 12 days of growth, filtered through a 0.2 µm pore-size cellulose acetate membrane filter (Sartorius Stedim Biotech, Göttingen, Germany) and mixed with CuCl_2_ solution at a 20 mM final concentration. The samples were placed on a roller shaker (60 r.p.m.) overnight, and precipitated products were collected and washed twice with Milli-Q water by centrifugation (×10000 ***g***, 30 min).

### Nanoparticle characterization

Morphological observations of the precipitated Cu nanoparticles were carried out using a Jeol-1200 EX transmission electron microscope (TEM) (Jeol, Welwyn Garden City, UK) and a Zeiss 710 confocal microscope (Zeiss, Jena, Germany). For the TEM analysis, about 10 mg nanoparticles was suspended in 1 ml Milli-Q water and 2 µl of the suspension solution was placed on ‘holey’ carbon-coated nickel grids (Agar Scientific, Essex, UK), and air dried prior to TEM analysis. For the confocal microscope analysis, the CuNPs sample was stained for 30 min at room temperature using 70 µM Fura-2AM (Abcam, UK) in the dark to visualize Cu, followed by staining using FilmTracer Sypro (Thermal Fisher, USA) to visualize proteins [32]. Stained samples were washed twice and resuspended using Milli-Q water, and 3 µl of resuspended samples were encased between a glass slide and a coverslip and sealed using nail varnish. The samples were imaged in real time using a Zeiss 710 confocal microscope with a 60× oil-immersion objective at room temperature.

### Degradation of methyl red in solution

Desired amounts of CuNPs and MR in 100 ml aqueous solution were placed in a 250 ml Erlenmeyer flask and kept at 25 °C in a shaking incubator at 125 r.p.m. The reaction was initiated by adding 30% H_2_O_2_ solution to give final concentrations of 2, 4 and 10 mM. In a typical degradation experiment, the amount of biogenic CuNPs in the reaction mixture was 20 mg and the concentration of MR was 15 mg l^−1^ (0.05 mM). The solution was adjusted to pH 7.5 by using 0.1M HCl and 0.1M NaOH. For the UV-Vis analysis, about 200 µl of the reaction solution was collected and scanned in a 96-well plate using a μQuant plate reader (Bioteck, Agilent Technologies LDA UK, Oxfordshire, UK). The concentration of MR in the reaction system at different times was calculated by measuring λ_max_ (425 nm) and calibrating from a standard curve.

### Cr(VI) removal from solution

CuNPs produced from biomass-free *N. crassa* spent growth culture supernatant and inorganic copper carbonate samples produced through the chemical reaction of 20 mM (NH_4_)_2_CO_3_ and 20 mM CuCl_2_ were used. Copper carbonate particle slurries were prepared at a concentration of 1.0 g l^−1^ total copper in water and Cr(VI) was added to an initial concentration of 200 mg l^−1^ K_2_CrO_4_. The solution for the adsorption experiments was adjusted to pH 7.5 using 0.1M HCl and 0.1M NaOH. Samples that were collected from solution at regular time intervals were immediately centrifuged (×10000 ***g***, 10 min) to stop further reaction. The supernatant was collected and the Cr concentration monitored using the 1,5-diphenylcarbazide assay (DPC) reaction [33], against a standard calibration curve for known Cr(VI) concentrations measured at a wavelength *λ*=540 nm.

### X-ray photoelectron spectroscopy (XPS)

XPS was performed to examine the CuNPs after Cr(VI) adsorption experiments using a Scienta ESCA-300 instrument (Scienta AB, Uppsala, Sweden) fitted with a non-monochromatic Al-Kα X-ray source. The survey spectra were collected from 1200 to 0 eV with a step size of 0.2 eV, and more detailed scans for the elements C, O, N and Cu were performed over the regions of interest. CasaXPS software was used to analyse XPS spectral curve fitting. All spectra were referenced to the O 1 s peak of carbonate at 530.9 eV.

### Cytotoxicity assay

Cultures of human cervical cancer cells (HeLa) were used to evaluate cytotoxicity of the CuNPs. Cells were grown in a Dulbecco’s Modified Eagle’s Medium (DMEM) with 10% FBS, and penicillin/streptomycin (100 units per ml) at 37 °C and 5% CO_2_. The cells were treated with various concentrations of CuNPs at 10, 20, 40, 60, 80 and 100 ng ml^−1^ at 37 °C and 5% CO_2_ for 24 h. The cells were then treated with the LD_50_ concentration of the CuNPs (40 ng ml^−1^), and samples were examined after 4, 8, 12, 16, 20 and 24 h of incubation. All treatments were performed at 37 °C and at a cell density allowing exponential growth. The DAPI assay was used to quantify fixed viable cells at 350/460 nm excitation/emission using a CLARIOstar plate reader (Kapuscinski 1995). Statistical analysis was performed using GraphPad Prism software with one-way analysis of variance and Tukey post-hoc tests for multiple comparisons. Values were considered significant at *P*<0.05.

## Results and discussion

### Formation and characterization of Cu carbonate nanoparticles (CuNPs) by transmission electron microscopy (TEM) and confocal microscopy

**Fig. 1.**
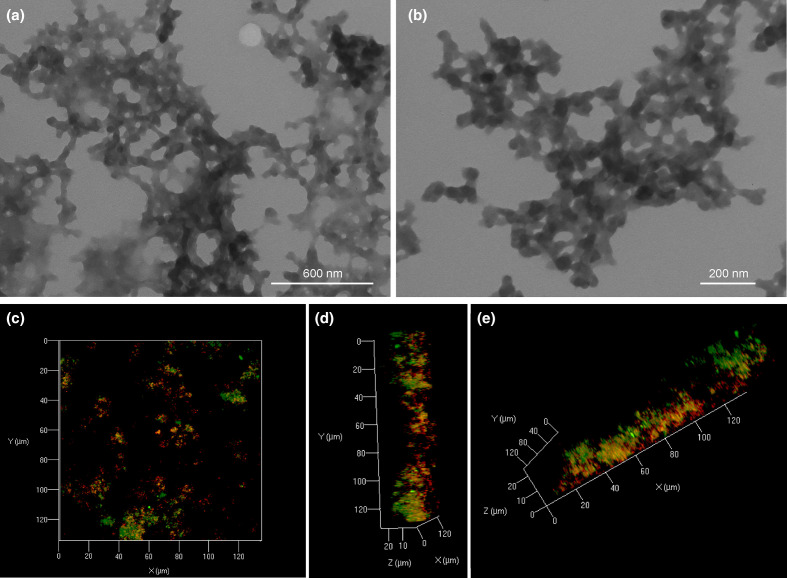
TEM (a, b) and confocal microscopy (c–e) images of CuNPs precipitated by mixing biomass-free *N. crassa* spent growth medium with 20 mM CuCl_2_. The confocal 3D data set was generated from 2D images visualizing the top, side and front view of the minerals, with proteins shown in red and Cu in green. Typical images are shown from several separate experimental determinations.

To optimize the formation of CuNPs and avoid precipitation of phosphate minerals, *N. crassa* was inoculated and incubated in an AP1 liquid medium for 3 days, followed by incubation in phosphate-free AP1 medium for 12 days at 25 °C. The fungal biomass was removed by centrifugation (×4000 ***g***, 30 min). Dark green minerals were precipitated and collected after mixing biomass-free *N. crassa* spent growth medium with 20 mM CuCl_2_ solution by centrifugation (×10000 ***g***, 30 min). TEM revealed the formation of particles with a size of around 20–40 nm (Fig. 1a, b). The confocal microscope images revealed the distribution of proteins (red) and copper (green) in the protein-nanoparticle aggregates, which confirmed a strong association of proteins with the CuNPs (Fig. 1c, e). The precipitated particles were amorphous, and the high amount of fungal proteins in the reaction system stabilized the amorphous copper carbonate during the early stages of mineral nucleation. The proteins present in the spent growth medium might enable the formation of a hydrogel-like system, which can organize the precipitated nanoparticles within a porous protein matrix. Similar structures have been observed for protein-mediated formation of some amorphous calcium carbonate (ACC) precursors [34].

### Methyl red (MR) degradation by CuNPs

Among a variety of pollutant remediation approaches, advanced oxidation processes (AOP) can offer a highly reactive, non-specific oxidation that enables the degradation of a wide range of organic pollutants in wastewaters [35]. The formation of strong oxidant hydroxyl radicals (HO·) by the Fenton reaction is one of the most powerful and simple catalytic technologies [36]. Fenton’s reagent includes a mixture of hydrogen peroxide and metal ions (e.g. Cu^2+^, Fe^2+^, Fe^3+^), and the formed hydroxyl radicals can effect the chemical decomposition of dyes by H-abstraction and addition of C–C unsaturated bonds [37]. Although many studies have focused on Cu-based Fenton catalysts [38–40], approaches to decrease the cost of the reactants (Cu^2+^ and H_2_O_2_) and increase reaction efficiency in a neutral or alkaline environment still need to be assessed.

**Fig. 2.**
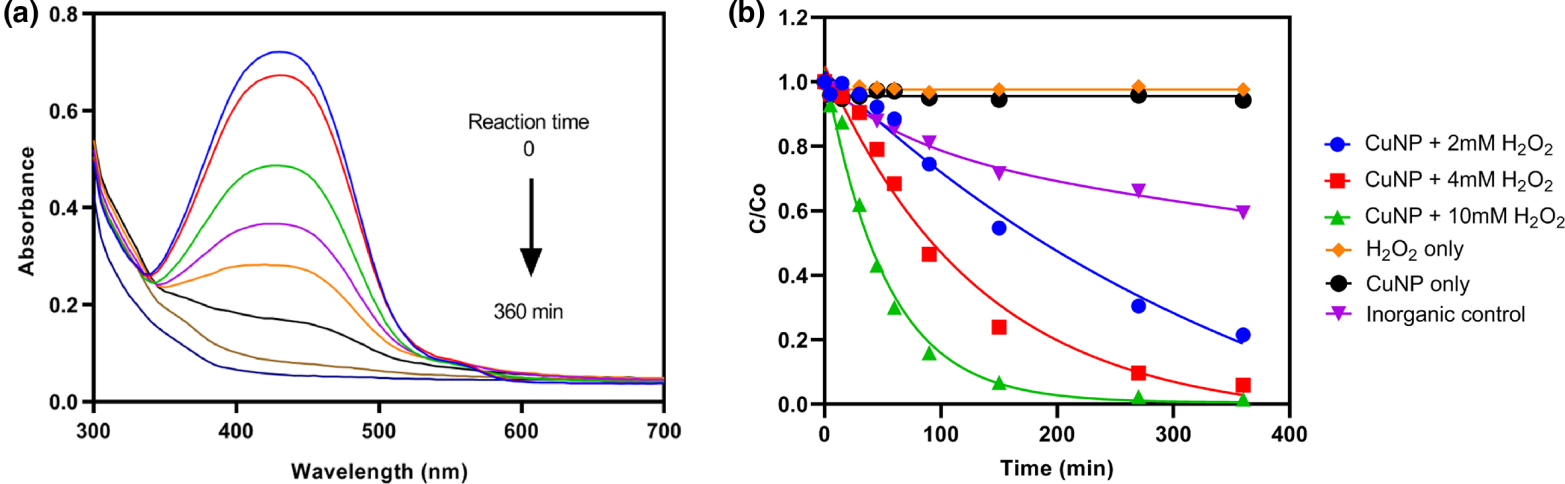
The degradation of MR by biogenic CuNPs. (a) UV-Vis spectral changes during the degradation of MR by CuNPs. 20 mg CuNPs and 10 mM H_2_O_2_ were added to 100 ml 15 mg l^−1^ MR solution, and the flasks were incubated at 25 °C, 125 r.p.m. in the dark. (b) Degradation of MR by CuNPs in the presence of various concentrations of H_2_O_2_. The inorganic control was used to assess the degradation potential of the biogenic CuNPs in comparison with inorganically synthesized copper carbonate, and the reaction was completed by adding 20 mg copper carbonate to 100 ml 15 mg l^−1^ MR solution in the presence of 10 mM H_2_O_2_. The concentration of MR at a given time point was measured as the absorbance at the maximum characteristic absorption wavelength (*λ*=425 nm). Experiments were carried out at least three times.

**Fig. 3.**
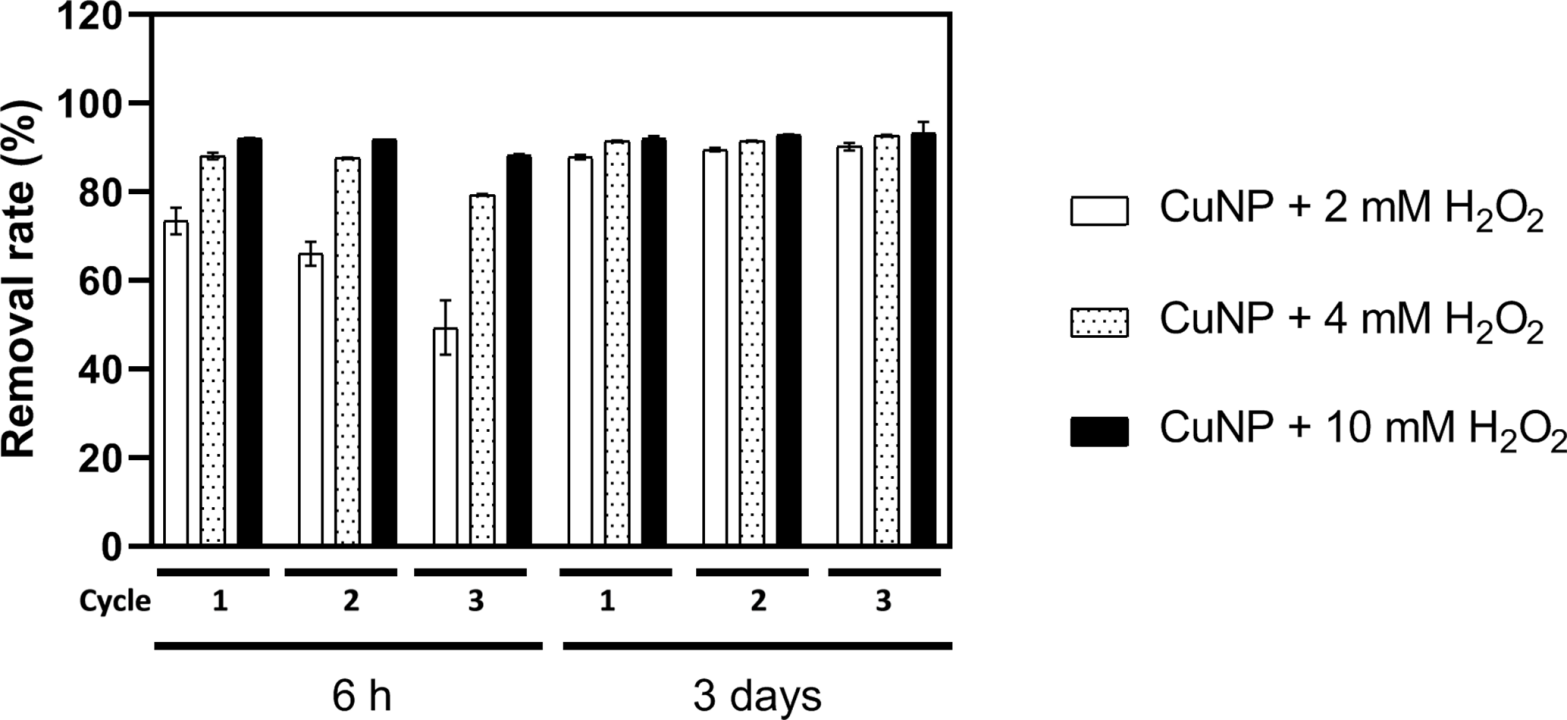
Reusability of catalyst CuNPs for MR degradation over three cycles of use. Reactions were carried out by adding 20 mg CuNPs and various concentrations of H_2_O_2_ into 100 ml 15 mg l^−1^ MR solution, and incubation at 25 °C, 125 r.p.m. in the dark. The values of MR in solution were measured after 6 h and 3 days for three cycles. Analyses were conducted in triplicate, and the bars shown are one standard error of the mean. The absolute value corresponding to 100% is 15 mg l^−1^. Samples are shown as CuNPs +2 mM H_2_O_2_, CuNPs +4 mM H_2_O_2_ and CuNPs +10 mM H_2_O_2_.

The removal of MR by CuNPs over time in a neutral environment (pH=7.5) is shown in Fig. 2. To monitor the MR degradation process, solution samples were harvested at different time intervals and the absorbance measured using a UV-Vis spectrophotometer. The MR solutions showed a significant absorbance peak around wavelength 425 nm at pH=7.5, and the change in the absorbance of MR in the presence of CuNPs and 10 mM H_2_O_2_ over time was visualized by scanning over the range 300–700 nm (Fig. 2a). MR is reported to show peaks at *λ*=520±15 nm and *λ*=435±20 nm, which are assigned to acidic and basic species of methyl red, respectively [41]. A gradual decrease in the absorbance values at wavelength 420 nm with time was observed, and the colour of the solution changed from brilliant red to colourless. The degradation of MR under different reaction conditions is shown in Fig. 2(b). The concentration of MR at selected time points was measured as the absorbance at the maximum characteristic absorption wavelength (*λ*=425 nm). The presence of aromatic groups in the MR structure makes it resistant to degradation, and almost no decrease in MR concentration was observed in a blank test. In the presence of only H_2_O_2_ or only CuNPs, the data also indicated there was almost no degradation of MR (Fig. 2b). This suggests that a direct reaction of H_2_O_2_ with MR, and adsorption of MR onto the CuNPs can be neglected in this MR removal process. However, in the presence of CuNPs and H_2_O_2_, rapid removal of MR was observed (Fig. 2b). The impact of the concentration of H_2_O_2_ on dye degradation was assessed, as the existence of an optimal concentration range has been reported in previous studies [42]. It can be predicted that additional H_2_O_2_ will not enhance contaminant degradation rates when the steady-state concentration of reactive oxygen species (ROS) remains unchanged in the system. In this study, the MR degradation rate increased with increasing H_2_O_2_ concentration and the reaction occurring in the presence of 10 mM H_2_O_2_ showed the highest value (Fig. 2b). Around 85% of the original MR was removed within 90 min, and more than 98% was degraded within 6 h. Nevertheless, even at a low concentration of 2 mM H_2_O_2_, >90% removal of MR was achieved after 3 days (Fig. 3). Inorganically synthesized copper carbonate, which was produced from mixture of 20 mM (NH_4_)_2_CO_3_ and 20 mM CuCl_2_ solutions, was used as a positive control to compare the different performance of biogenic CuNPs with inorganic copper carbonate. After 6 h reaction in the presence of 20 mg inorganically synthesized copper carbonate and 10 mM H_2_O_2_, around 40% of the dye was removed from solution. Under the same experimental conditions, the removal value for biogenic CuNPs was more than 98%. Therefore, there is no doubt that the biogenic CuNPs showed a much higher potential regarding MR remediation. The inorganically synthesized copper carbonate was characterized as malachite (Cu_2_(OH)_2_CO_3_), showing as highly aggregated spherical minerals with a size ranging from 0.5 to 5 µm in diameter [31]. In this case, the higher specific surface area of the biogenic nanoparticles would explain their higher reactivity with dye degradation when compared with larger inorganically produced particles.

Reusability is a desired attribute of a catalyst in practical industrial applications. The reusability of biogenic CuNPs was examined by repeated use for catalytic MR degradation. From one degradation cycle to the next, the CuNPs were recovered by centrifugation (×10000 ***g***, 10 min) and washed with distilled water to remove possible dye residues. Fig. 3 shows that there was no obvious deactivation of the CuNPs in the presence of 10 mM H_2_O_2_ after three cycles. The degradation rate became slower with increasing number of experimental cycles when the H_2_O_2_ concentration was low (2 mM), as only around 50% MR was removed within 6 h during the third cycle. Nevertheless, no obvious difference in MR degradation by CuNPs was found when the reaction time was 3 days, and the removal rate reached around 95% for all tests after three cycles, regardless of the initial concentration of H_2_O_2_ (Fig. 3). This indicated that the CuNPs' catalyst had excellent long-term stability under neutral conditions.

The degradation kinetics of MR were studied for various contact times ranging from 5 min to 6 h. The data were regressed based on first-order kinetic (Equation 1), pseudo-first-order kinetic (Equation 2) and second-order kinetic equations (Equation 3).

Equation (1):

$$lnC_{t}=lnC_{0}-k_{1}t$$

Equation (2):

$$\log\left(C_{t}-C_{e}\right)=\log\left(C_{0}-C_{e}\right)-\left(\frac{k_{1}}{2.303}\right)t$$

Equation (3):

$$\frac{1}{C_{t}}=\frac{1}{C_{0}}+k_{2}t$$

where *C*_0_ is the initial concentration of MR, *C*_*t*_ is the concentration of MR at time *t*, and *C*_*e*_ is the equilibrium absorbance. *k*_1_ (min^−1^) and *k*_2_ (C^−1^min^−1^) are the rate constants of the first-order and second-order kinetic equations, respectively, which can be calculated from the slopes and intercepts of the linearized models. A comparison of three kinetic models for the experimental results and the calculated kinetic parameters obtained from curve fitting are presented in Table 1. The first-order and second-order kinetics did not fit the data well as indicated by the lower regression coefficients. The linearized pseudo-first-order model provided much better *R* ^2^ values than those for other models (Fig. S1, available with the online version of this article). The decolourization of MR by CuNPs therefore appeared to follow pseudo-first-order kinetics. The rate constants increased and half-lives decreased gradually with an increase of H_2_O_2_ concentration in the reaction system, which is consistent with the experimental data.

**Table 1.** Kinetic constants for the degradation of MR by CuNPs in the presence of various concentrations of H_2_O_2_

H_2_O_2_ (mM)	First-order kinetics			Pseudo-first-order kinetics			Second-order kinetics		
	*k*_1_ (min^−1^)	*T*_1/2_ (min)	*R* ^2^	*k*_1_ (min^−1^)	*T*_1/2_(min)	R^2^	*k*_2_ (C^−1^ min^−1^)	*T*_1/2_(min)	*R* ^2^
2	0.0045	154.0	0.9906	0.0189	130.9	0.9895	0.0006	108.7	0.9591
4	0.0084	82.5	0.9879	0.0097	71.7	0.9938	0.0028	23.3	0.9456
10	0.0122	56.8	0.9436	0.0053	36.7	0.9945	0.0112	5.8	0.9623

The degradation of a series of dyes, including methyl orange, methyl blue and rhodamine B by Cu-bearing nanoparticles has been reported previously. In this Fenton-like reaction, Cu^2+^ of the Cu nanoparticles can be reduced by H_2_O_2_, which will be further oxidized with the formation of highly ROS HO· [43]:

Equation (4):

$$Cu^{2+}+H_{2}O_{2}⟶Cu^{+}+HO_{2}\cdot +OH^{-}$$

Equation (5):

$$Cu^{+}+H_{2}O_{2}⟶Cu^{2+}+HO\cdot +OH^{-}$$

The organic dyes will then be decomposed due to the high oxidizing ability of HO·. This approach is considered to be a process of high efficiency and produces minimal secondary pollutants. Sun *et al*. [43] studied dye degradation by Cu_2_O−Cu/C catalysts. The degradation of methyl orange, methyl red and toluidine blue with copper(II) ions in a H_2_O_2_/HCO_3_^−^ solution has also been reported [40]. These results showed the formation of a CuCO_3_ complex, which was probably more active than the other copper species and responsible for the effective dye removal. A more detailed characterization of the biogenic CuNPs produced from the biomass-free *N. crassa* spent growth medium has been reported in earlier work [31]. Fourier-transform infrared spectroscopy (FTIR) and XPS revealed these fungal synthesized CuNPs were primarily composed of copper carbonate, which can be a precursor for other Cu-bearing compounds such as CuO. In this study, we also compared the catalytic activity of the unaltered biogenic CuNPs with Cu oxides derived from thermal transformation of the CuNPs (see Figs S2 and S3). After a one-step thermal transformation process in a muffle furnace at 975 °C for 30 min, the incinerated products of biogenic CuNPs were collected and examined. The diffraction peaks in the XRD patterns of the carbonized products can be assigned to CuO (around 81.2%) and Cu_2_O (around 18.8%). Compared with the biogenic CuNPs, the degradation of MR in the presence of the Cu oxides and 10 mM H_2_O_2_ showed a much slower reaction rate, with only 53% of MR being degraded within 6 h. Furthermore, the catalytic activity of the Cu oxide materials decreased significantly in the reusability test. Only 13.4% of MR was degraded within 6 h when the Cu oxides were reused for the third time, while the biogenic CuNPs showed more than 90% of MR removal under the same conditions. All these results suggest the ‘green’ synthesis of copper carbonate nanoparticles from fungal spent culture medium could supply a promising alternative method for production of Cu-bearing NP catalysts, with benefits of easy synthesis and high reusability.

### Cr (VI) removal of by biogenic CuNPs

**Fig. 4.**
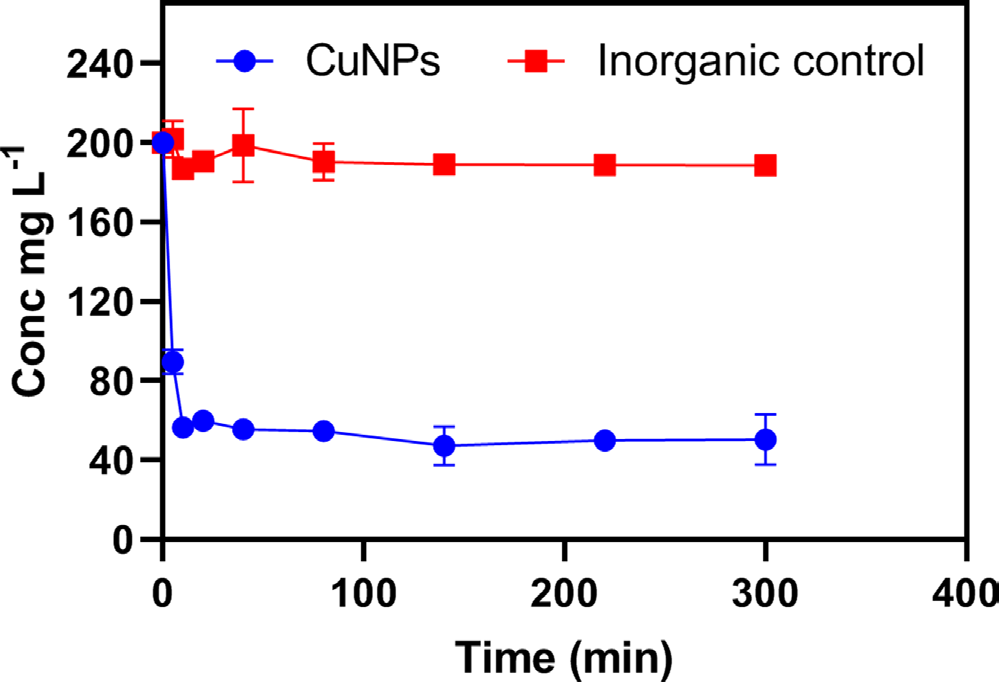
Cr(VI) removal by biogenic CuNPs and inorganic copper carbonate at 1 g l^−1^ total Cu. The concentration of Cr in solution was measured by the DPC reaction method. Analyses were conducted at least in triplicate, and the bars shown are one standard error of the mean.

The reactivity of biogenic CuNPs was also measured by their ability to remove Cr(VI) from solution. A biogenic CuNP suspension was mixed with 200 mg l^−1^ Cr(VI) with a Cu loading of 1 g l^−1^, and the concentration of Cr(VI) in the solution was measured by the DPC reaction method. Compared to the control sample produced from the inorganic synthesis of copper carbonate through the chemical reaction of 20 mM (NH_4_)_2_CO_3_ and 20 mM CuCl_2_ solutions, which showed little removal of Cr (VI), the biogenic CuNPs removed around 75% Cr(VI) from the solution within 5 h (Fig. 4). The removal of Cr(VI) from solution was rapid and reaction equilibrium was achieved within 40 min, with the final concentration of Cr in the solution ~50 mg l^−1^. The CuNPs produced by biomineralization therefore showed a higher capability for Cr(VI) removal compared to inorganically synthesized copper carbonate, which was probably due to their higher specific surface area and Cr(VI) reduction by reducing-Cu species. The high large surface area to volume ratio of nanoparticles imbue them with high reactivity, and the capability to detoxify or immobilize pollutants [2]. Previous studies have also pointed out the chromate reductase activity of some fungal cytosolic or membrane proteins, even in cell-free extracts, which may contribute to fungal Cr(VI) resistance and Cr(VI) reduction [44, 45]. The biogenic CuNPs formed from biomass-free growth medium of *N. crassa* were associated with various fungal proteins, and it is plausible that fixed proteins on the nanoparticle surface can exhibit some activity [46], and participate in the Cr(VI) reduction process. In future work, the impact of fungal proteins on Cr(VI) reduction could be investigated by studying the interaction of Cr(VI) with fungal growth medium (i.e. no CuNPs), and/or specific purified proteins of interest, which could clarify the relative roles of Cu and protein in Cr(VI) remediation.

**Fig. 5.**
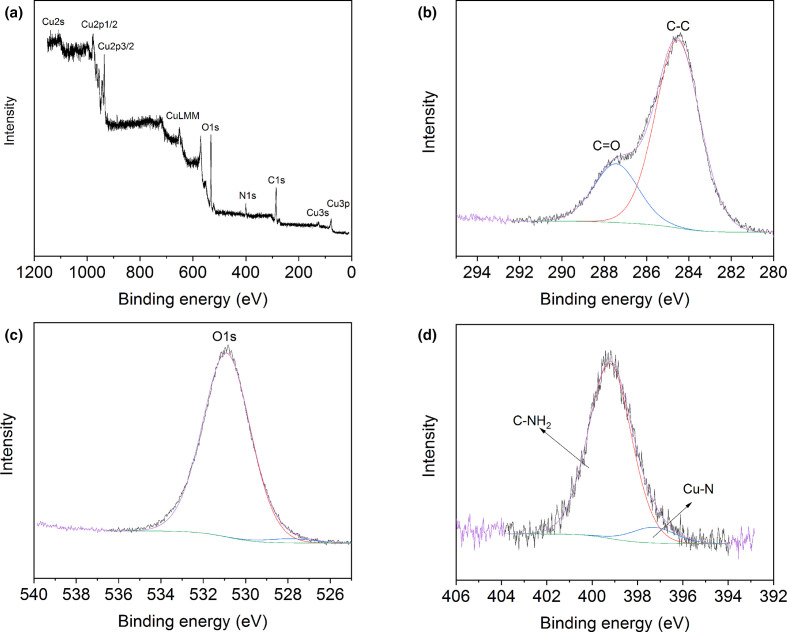
XPS spectra of CuNPs after Cr(VI) adsorption. (a) Total survey showing the presence of Cu, O, C and N on the surface of CuNPs; (b–d) High-resolution XPS spectra of C1s, O1s and N1s. Typical spectra are shown.

**Fig. 6.**
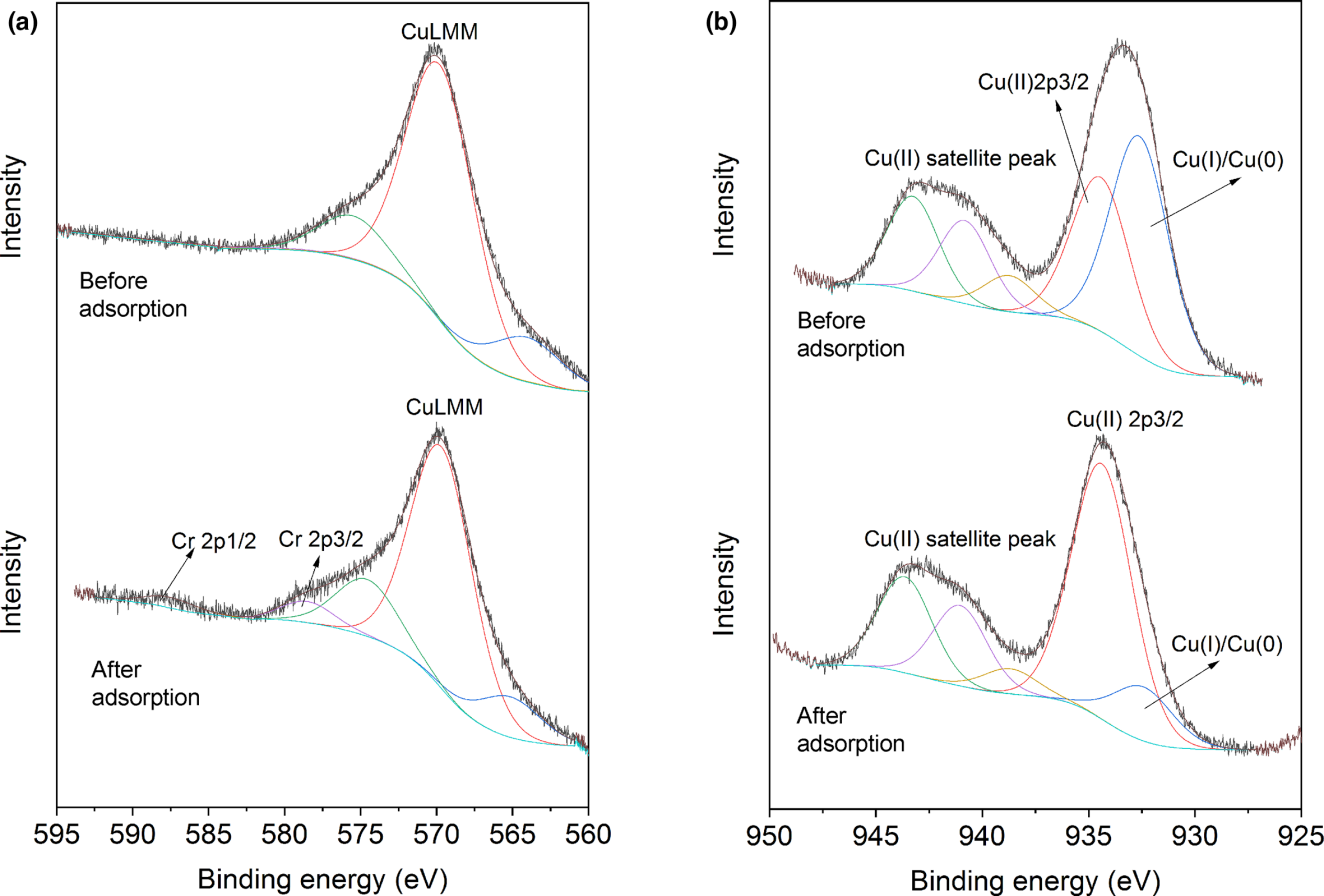
XPS spectra of CuNPs CuLMM and Cu2p3/2 before and after Cr(VI) adsorption. (a) Cu LMM spectrum showing the signals of Cr 2p3/2 and Cr 2p1/2; (b) Cu2p3/2 spectrum (upper spectrum before reaction with Cr(VI), adapted from [31]) showing the oxidation of Cu species after the reaction of CuNPs with Cr(VI) solution (lower spectrum).

After adsorption of Cr(VI), no new Cr-bearing mineral phases were detected by XRD. The elemental composition of the CuNPs surface and adsorption of Cr by the CuNPs were further analysed by XPS. Distinct peaks of C (1 s), O (1 s), N (1 s) and Cu were observed (Fig. 5). The C 1 s spectra were deconvoluted into two Lorentzian–Gaussian peaks, identified as the C–C bond at 284.6 eV, and the C=O bond of carbonate groups at 287.5 eV. The N 1 s spectra could also be deconvoluted into two peaks, indicating the presence of amide C–NH_2_ (399 eV) and complexes of Cu–N (397.4 eV). Compared with the original CuNPs surface, no obvious changes in the N, C and O spectra were detected after Cr(VI) adsorption. The occurrence of Cr peaks on the CuNPs' surface was confirmed from the high-resolution XPS spectra after adsorption, as shown in Fig. 6(a). As the Cr peaks were highly overlapped by the CuLMM peak, further deconvolution to clarify the redox status of Cr was not possible. Nevertheless, the spectra of Cu 2p3/2 showed a significant decrease in the amount of Cu(I) and/or Cu(0) species after the reaction with Cr(VI) (Fig. 6b). The organic molecules that may be present in the fungal growth supernatant, such as some cysteine-rich proteins, have been reported to be able to reduce Cu (II) leading to the formation of Cu(I)-bearing complexes [47]. The decrease in the signals for reduced Cu species suggested that the Cu(I) and/or Cu(0) of the CuNPs participated in Cr(VI) reduction, resulting in the formation of oxidized Cu(II) and the probable formation of less toxic Cr(III) on the surface of CuNPs.

The removal of Cr(VI) by Cu-based nanoparticles has been reported in some previous studies. A CuPd nanocatalyst was employed for the reduction of toxic Cr(VI) to less toxic Cr(III) at room temperature [48]. Copper carbonate nanoparticles (malachite) were shown to be a potential nanomaterial for toxic pollutant (e.g. Cr and arsenate) remediation through adsorption [22]. Nano zero-valent iron/Cu nanoparticles synthesized using green tea extract also showed effective Cr(VI) removal from solution through co-precipitation and adsorption [49]. Co-precipitation refers to the interaction of Cu and Cr and the subsequent precipitation of materials consisting of both copper and chromium with a defined stoichiometric relationship. As Cr(VI) is not able to precipitate in the forms of carbonate or hydroxylate minerals, Sun and Huang [50] studied the co-precipitation of Cr(VI) during copper carbonate precipitation in a batch reactor containing both Cr and Cu. The addition of carbonate ions triggered the formation of Cu–Cr minerals, and their results suggested precipitation of copper carbonate was always accompanied by some chromium removal through the formation of CuCrO_4_ crystallites and adsorption [50, 51]. By studying the mechanisms of reaction of Cu carbonate with Cr(VI), this might shed some light on improving the co-removal of toxic metals from contaminated wastewater, either by a chemical approach or by using microbial-induced mineral precipitation.

### Cytotoxicity of CuNPs

**Fig. 7.**
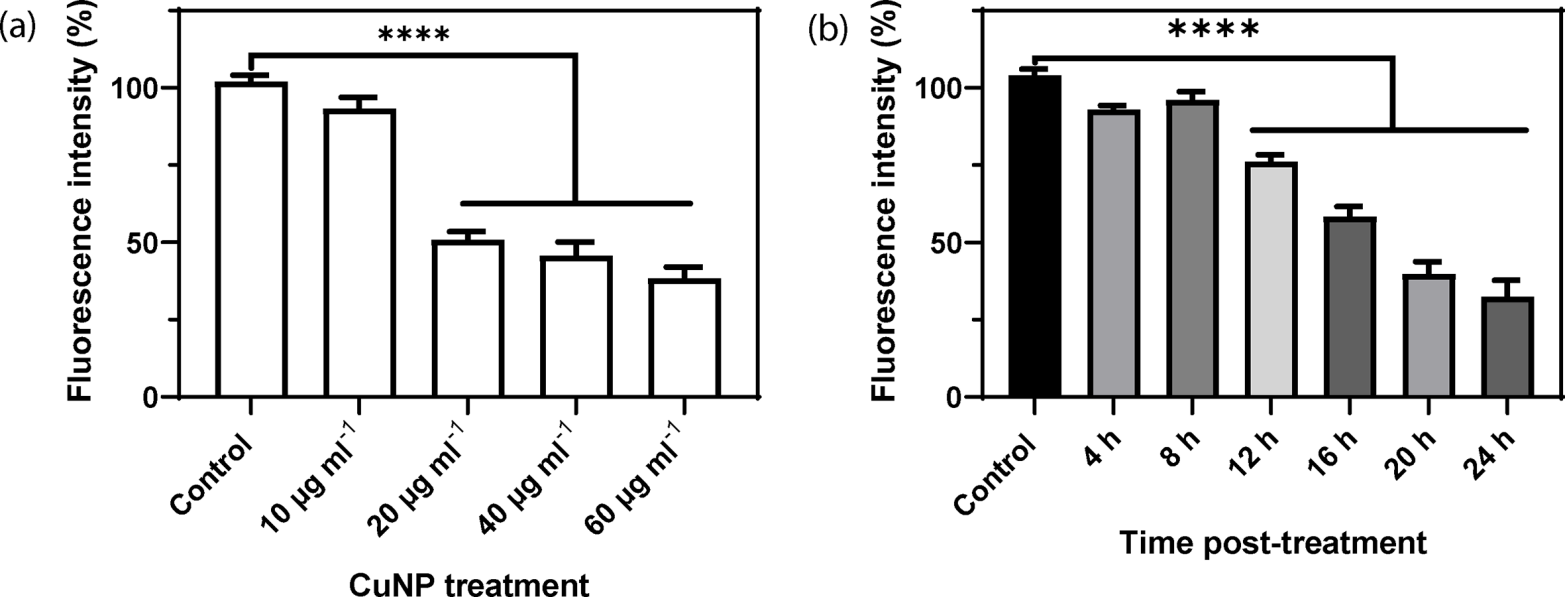
Cytotoxicity of CuNPs examined using HeLa cells. (a) Treatment with a CuNP concentration over 40 μg ml^−1^ caused a significant decrease in cell number compared with the untreated control. (b) When cells were treated with 40 μg ml^−1^ CuNP for 12 h or more, a significant decrease in cell viability was observed compared with the untreated control. Asterisks indicate a significant change (*P*<0.0001) from at least six experimental determinations.

The *in vitro* cytotoxic study using Hela Cells was conducted by examining changes in cell density after exposure to different concentrations of the biogenic CuNPs harvested from fungal growth medium. HeLa cells were treated with various concentrations of CuNPs (0–100 ng ml^−1^) for 24 h, and cell survival was determined using a DAPI assay (Fig. 7). Cell viability decreased with increasing CuNPs concentration, with the LD_50_ being around 40 μg ml^−1^. When cells were treated with the LD_50_ concentration, cell viability decreased significantly after 12 h. The development and application of engineered nanoparticles in various fields have been the subject of increasing concern due to their possible implications for human health and ecosystem function. Various copper-containing nanoparticles such as CuO, amorphous and crystalline CuS and Cu phosphate have been reported to suppress proliferation and viability of several cell lines, including various mouse and human cells [52, 53]. Copper-based nanoparticles are reported to induce oxidative stress in cells, which further induces DNA damage, mitochondrial membrane damage, and apoptosis [52, 54]. How biogenic CuNPs can lead to ROS generation, induce DNA damage, and perturb mitochondrial membrane potential followed by apoptosis requires study in the future. The potential toxicity of various Cu-bearing minerals, including abiotically synthesized and biogenic Cu-containing nanoparticles, is an appropriate subject for future research.

The small size and catalytic properties of Cu make biogenic CuNPs potentially very suitable for a wide variety of applications. This research has demonstrated the catalytic activity of biogenic CuNPs, formed from biomass-free spent *N. crassa* ureolytic growth medium, for methyl red degradation and chromate remediation. A rapid and complete decolourization of methyl red in solution occurred in the presence of CuNPs and H_2_O_2_. Cu can react with H_2_O_2_ and form highly ROS, which can oxidize and decompose organic dyes in solution. No obvious deactivation of the CuNPs were found, even after three cycles of operation, which confirmed the reusability of the CuNPs catalyst. The removal of Cr(VI) utilized the high-adsorption capability of the nanoparticles with a high surface area to volume ratio coupled with reactive Cu(I)/Cu(0) on the surface of CuNPs. It is likely that reducing Cu species mediated the reduction of highly toxic Cr(VI) to less toxic Cr(III), although this would require further detailed analysis of Cr speciation. The precipitation of CuNPs by *N. crassa* also sheds light on sustainable Cu biorecovery from Cu-enriched waste streams. Instead of using Cu salts to synthesize Cu nanoparticles with catalytic efficiency, the potential bioprecipitation or biorecovery of CuNPs from waste streams through fungal-mediated carbonate precipitation could provide a better solution in terms of toxic metal remediation, and even the co-removal of toxic metals.

## Supplementary Data

Supplementary material 1
