# Supplementary material for: Application of fungal copper carbonate nanoparticles as environmental catalysts: organic dye degradation and chromate removal
Source: Microbiology (Reading). 2021 Dec 9;167(12):001116. doi: 10.1099/mic.0.001116 (PMC8745000; doi:10.1099/mic.0.001116)
Supplement: Supplementary material 1 [file mic-167-1116-s001.pdf]

Supplementary Material

Application of fungal copper carbonate nanoparticles as environmental catalysts: organic dye degradation and chromate removal

Feixue Liu,[1] Dinesh Singh Shah,[2] Laszlo Csetenyi,[3] Geoffrey Michael Gadd [1,4,5 *]

[1] Geomicrobiology Group, School of Life Sciences, University of Dundee, Dundee, DD1 5EH, Scotland, United Kingdom

[2] Division of Cell Signalling and Immunology, School of Life Sciences, University of Dundee, Dundee, DD1 5EH, Scotland, United Kingdom

[3] Concrete Technology Group, Department of Civil Engineering, University of Dundee, Dundee, DD1 4HN, Scotland, United Kingdom

[4] State Key Laboratory of Heavy Oil Processing, Beijing Key Laboratory of Oil and Gas Pollution Control, College of Chemical Engineering and Environment, China University of Petroleum, Beijing 102249, China

[5] Lead Contact

[*] Correspondence: g.m.gadd@dundee.ac.uk (G.M.G)

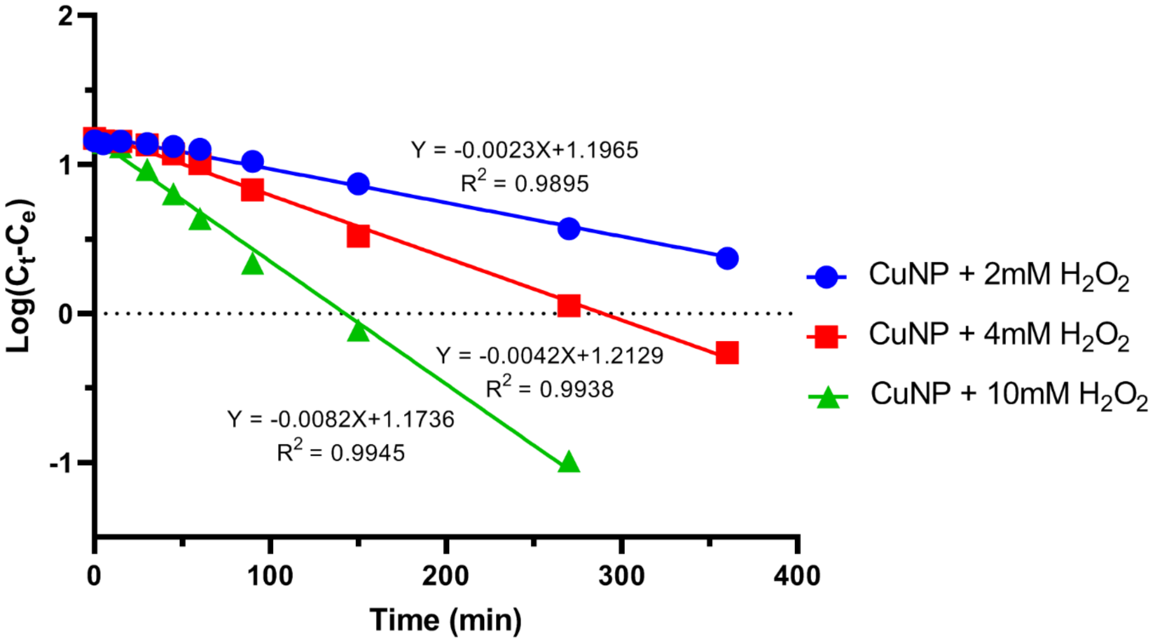

Figure S1 Linearized pseudo-first-order kinetic models.

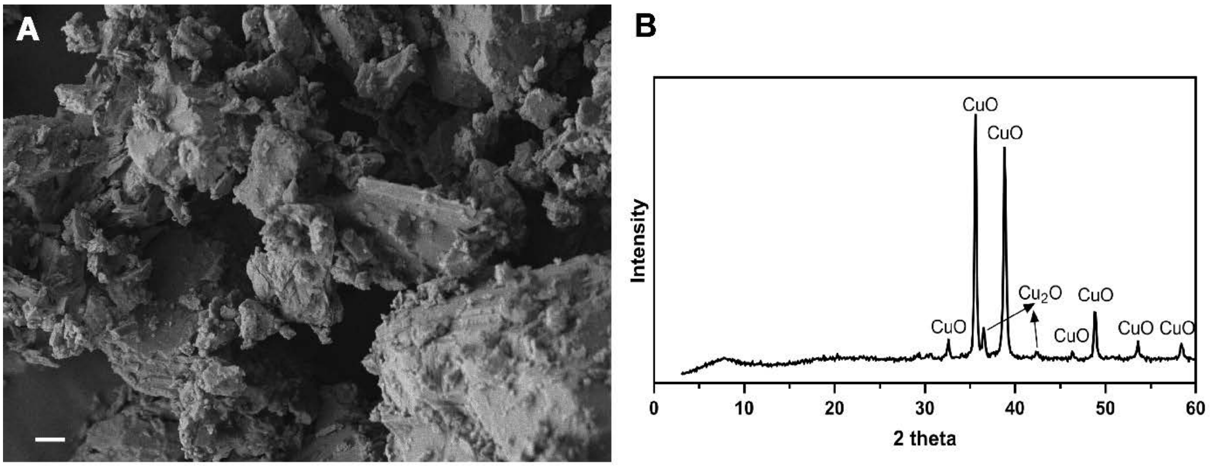

Figure S2 (A-B) Characterization of thermal decomposition product of CuNPs. (A) SEM images of the Cu-bearing minerals, with the scale bar being 1 μm; (B) XRD pattern showing the formation of CuO with a small amount of Cu_2O. Typical image and XRD pattern are shown from several separate determinations.

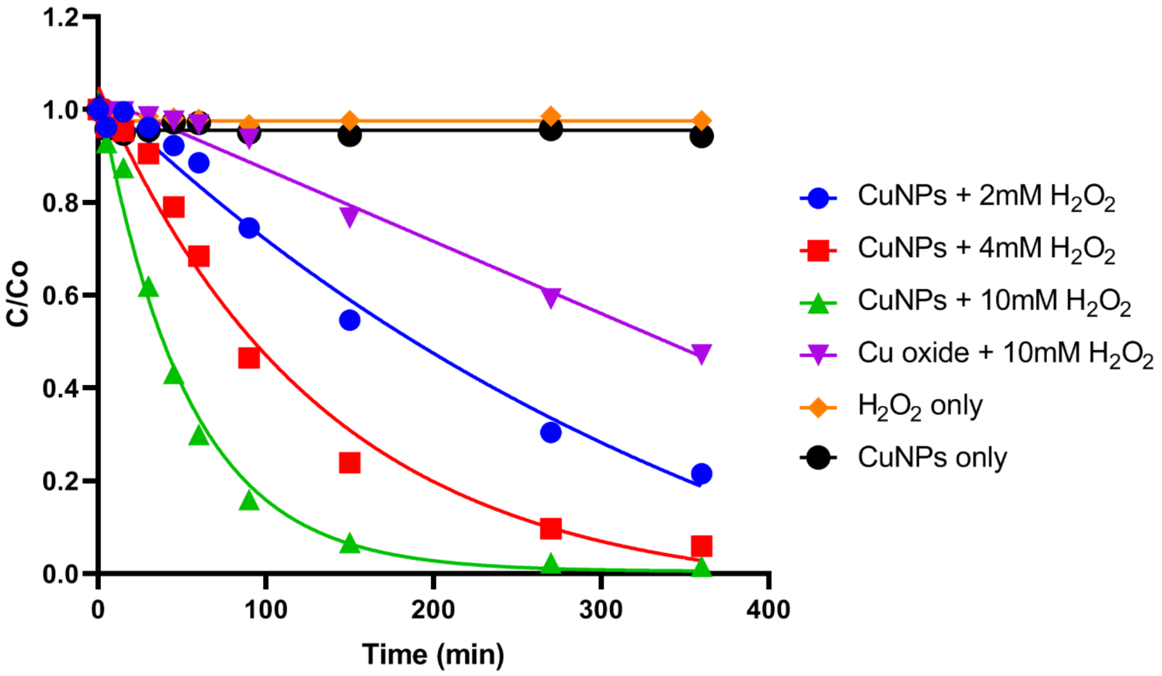

Figure S3 The catalytic activity of Cu oxide produced from thermal decomposition of CuNPs tested through the degradation of MR.
